# The use of exenatide in severely burned pediatric patients

**DOI:** 10.1186/cc9222

**Published:** 2010-08-11

**Authors:** Gabriel A Mecott, David N Herndon, Gabriela A Kulp, Natasha C Brooks, Ahmed M Al-Mousawi, Robert Kraft, Haidy G Rivero, Felicia N Williams, Ludwik K Branski, Marc G Jeschke

**Affiliations:** 1 Department of Surgery, University of Texas Medical Branch, 301 University Blvd., Galveston, Texas 77555, USA; 2 Shriners Hospitals for Children, 815 Market Street, Galveston, Texas 77550, USA

## Abstract

**Introduction:**

Intensive insulin treatment (IIT) has been shown to improve outcomes post-burn in severely burnt patients. However, it increases the incidence of hypoglycemia and is associated with risks and complications. We hypothesized that exenatide would decrease plasma glucose levels post-burn to levels similar to those achieved with IIT, and reduce the amount of exogenous insulin administered.

**Methods:**

This open-label study included 24 severely burned pediatric patients. Six were randomized to receive exenatide, and 18 received IIT during acute hospitalization (block randomization). Exenatide and insulin were administered to maintain glucose levels between 80 and 140 mg/dl. We determined 6 AM, daily average, maximum and minimum glucose levels. Variability was determined using mean amplitude of glucose excursions (MAGE) and percentage of coefficient of variability. The amount of administered insulin was compared in both groups.

**Results:**

Glucose values and variability were similar in both groups: Daily average was 130 ± 28 mg/dl in the intervention group and 138 ± 25 mg/dl in the control group (*P *= 0.31), MAGE 41 ± 6 vs. 45 ± 12 (respectively). However, administered insulin was significantly lower in the exenatide group than in the IIT group: 22 ± 14 IU patients/day in the intervention group and 76 ± 11 IU patients/day in the control group (*P *= 0.01). The incidence rate of hypoglycemia was similar in both groups (0.38 events/patient-month).

**Conclusions:**

Patients receiving exenatide received significantly lower amounts of exogenous insulin to control plasma glucose levels. Exenatide was well tolerated and potentially represents a novel agent to attenuate hyperglycemia in the critical care setting.

**Trial registration:**

NCT00673309.

## Introduction

Hyperglycemia is a common finding in critically ill patients that has been associated with increased morbidity and mortality. In burns, it has also been shown that hyperglycemia is deleterious. Gore *et al*. [1] found that hyperglycemia was associated with an increased rate of muscle protein catabolism [2] and increased morbidity and mortality in this population. In addition, Hemmila *et al*. [3] found that IIT was associated with a lower incidence of pneumonia, ventilator-associated pneumonia and urinary tract infections; and Pham *et al*. [4] reported similar findings and a positive association of IIT with survival rates following IIT treatment in pediatric burned patients. Our group has recently shown that IIT in severely burned pediatric patients was associated with improved post-burn morbidity, such as infection, sepsis, and organ function [5].

However, the Normoglycemia in Intensive Care Evaluation and Survival Using Glucose Algorithm Regulation (NICE-SUGAR) found no benefit and an increased incidence of hypoglycemia with IIT in adult critical care [6,7]. However, detailed analysis of the NICE-SUGAR study suggests a better outcome within the trauma subpopulation with intensive insulin treatment [6].

Since current evidence supports the use of IIT in trauma patients, the study of novel therapies to decrease hyperglycemia in burn patients without increasing the risk of hypoglycemia is warranted [8]. Incretin-based therapies are currently among the newest classes of available glucose-lowering agents [9]. The incretin effect consists of higher insulin production after an oral ingestion of glucose than after an intra-venous load one [10]. The incretins that have been identified are glucose-dependent insulinotropic peptide (GIP) and glucagon-like peptide-1 (GLP-1). Exogenous GLP-1 has been shown to reduce glucose concentration when administered to hospitalized patients [11,12]. Activation of the incretin receptors on β-cells increases insulin release in response to glucose [13] and may have additional beneficial effects, as it has been suggested that these drugs promote enhanced glucose disposal in peripheral tissues and protect against ischemia/reperfusion injury [14].

Exenatide is a synthetic peptide originally identified in the lizard *Heloderma suspectum *[15] that possesses incretin-mimetic actions including suppression of glucagon secretion and delay of gastric emptying [16]. It has been shown to bind and activate the human glucagon-like peptide-1 (GLP-1) receptor *in vitro *[17]. We hypothesized that exenatide would decrease the amount of exogenous insulin administered to the patients and the incidence of hypoglycemia in the acute setting of severely burned pediatric patients.

## Materials and methods

### Patients

Twenty-four severely burned pediatric patients were recruited for the study. This study was approved by the Institutional Review Board of The University of Texas Medical Branch. The patient, parent or legal guardian signed an informed consent for this study.

### Medical care

Medical care was determined by faculty surgeons, fellows, and residents according to clinical protocols that have been previously described [18]. Briefly, patients were fed with Vivonex^® ^T.E.N. (total enteral nutrition). (Novartis, Minneapolis, MN, USA; 82% carbohydrate, 15% protein, 3% fat, glutamine 4.9 g/L and L-Arginine 2.9 g/L) at 1.4 times their measured resting energy expenditure (REE) [4] and initiated within 48 h after a burn injury. The nutritional route of choice in our patient population was enteral nutrition via a duodenal (Dobhof) tube. The patients received nutritional supplements including multivitamin (Enfamil Poly-Vi-Sol ^®^, Mead Johnson & Company, LLC, Evansville, IN USA) 29.5 ml (1 fl oz) per os (PO) daily; folic acid 1 mg PO three days a week; zinc sulfate PO 55 mg for patients under 2 years old, 110 mg for patients 3 to 11 years old and 220 mg for patients older than 12 years old; and vitamin C 250 mg PO for patients under 12 years old and 500 mg for patients 12 years old and older. Burn patients were excluded from participation if they were diagnosed with diabetes mellitus before the burn injury. We used oral glucose tolerance tests, medical history and determinations of glycosylated hemoglobin (HbA1C) to detect diabetic patients. Potential side effects of GLP-1, such as gastrointestinal symptoms (for example, nausea, pyrosis), injection site reactions and hypersensitivity symptoms, were prospectively evaluated and documented.

### Indirect calorimetry

As part of our routine clinical practice, all patients underwent REE measurements within one week following hospital admission and weekly thereafter during their acute hospitalization. All measurements of REE were performed between midnight and 5 a.m. while the patients were asleep and receiving continuous feeding. REE was measured using a Sensor-Medics Vmax 29 metabolic cart (Yorba Linda, CA, USA) calibrated according to the manufacturer instructions as previously published [19]. The REE was calculated from the oxygen consumption and carbon dioxide production by equations described by Weir *et al*. [20]. Measured values were compared to predicted norms based upon the Harris-Benedict equation [21] and to body mass index (BMI). For statistical comparison, energy expenditure was expressed as the percentage of the basal metabolic rate predicted by the Harris-Benedict equation.

The patients in this study were mechanically ventilated only for operative procedures and there was no difference in mechanical ventilation between both groups.

### Treatment

Participants were randomly assigned (block randomization 1:3) during their acute hospitalization (until 95% healed) to exenatide treatment and insulin as an adjunct if needed, or only intensive insulin. Patients assigned to exenatide received exenatide (Amylin Pharmaceuticals, Inc., San Diego, CA, USA) subcutaneous (SQ) and insulin, if needed, to maintain plasma glucose levels between 80 and 140 mg/dl.

Exenatide was adjusted to plasma glucose levels. It was initiated with 5 μg of exenatide q 12 h increasing the dose to 10 μg up to q 4 h if glucose levels were above target. If the glucose was still above target, we then added insulin therapy as described below.

The intensive insulin treatment (IIT) patients were treated with intensive insulin to maintain plasma glucose levels between 80 and 140 mg/dl.

Regular insulin was administered in a sliding scale to titrate to 80 to140 mg/dl. Infusion started at a rate of 0.1 U/kg/h, with increments ranging from 0.1 U/kg/h for glucose 141 to 160 mg/dl up to 1 U/kg/h when 1 U/kg/h when glucose was greater than 260 mg/dl.

### Laboratory

Blood samples for glucose determination were obtained during the acute hospitalization and analyzed in the laboratory of the hospital. In all the patients, glucose levels were measured in a panel with liver enzymes and electrolytes. Insulin levels were determined by ELISA. Initially, and if any change in insulin infusion or feeding occurred or if glucose levels were not in the desired range, glucose was checked every 15 minutes until stable (defined as three consecutive measurements with glucose on range). Once stable, determinations were reduced every two hours. Routine glucose determinations were performed on a daily basis at 06:00 h.

In all the patients with at least three glucose determinations, the daily average, maximum and minimum glucose levels were determined. For those patients with one to two glucose values per day, only 6 AM glucose was used for calculations of daily average. A hypoglycemic event was defined as plasma glucose level < 60 mg/dl preceded by at least two normal values.

Mean amplitude of glucose excursion (MAGE) and percentage of coefficient of variance (% CV) were used to assess variability of glucose values. MAGE assesses the average amplitude of upstrokes and downstrokes with magnitude greater than one standard deviation [22]. The %CV is defined as: %CV = 100*SD/mean [23].

### Statistical analysis

We used the Mann-Whitney U test and Chi square analysis. Data are expressed as means ± SD or SEM, where appropriate (SigmaStat v3.5.1.2 Heame Scientific Software, Chicago, IL, USA). Significance was accepted at *P *< 0.05.

## Results

**Figure 1:**
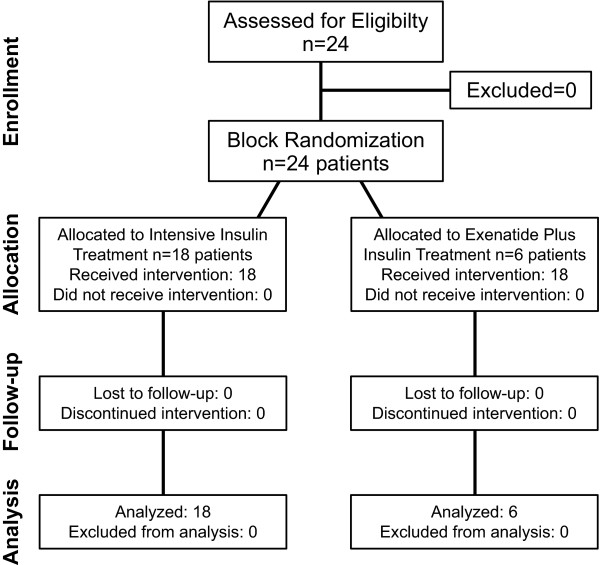
**CONSORT diagram**. Eligibility and enrollment of patients.

Six patients were randomized to the treatment group and 18 patients to the IIT group (Figure 1). Demographics showed no significant difference between both groups (Table 1). Glucose values were similar in both groups (Table 2). The number of glucose determinations was similar between both groups: 143 ± 38 for the exenatide group and 139 ± 59 for the IIT group (*P *= 0.79).

**Table 1:** Demographics

Demographics	IIT	Exenatide	*P *value
Age (years)	12 ± 4	12 ± 5	NS
Gender (M:F)	(3.5:1)	(5:1)	NS
Weight (kg)	41 ± 19	49 ± 23	NS
Height (cm)	145 ± 28	144 ± 26	NS
TBSA (%)	61 ± 16	56 ± 13	NS
3^rd ^(%)	51 ± 25	46 ± 19	NS
Type of burn			
Flame	67%	83%	NS
Scald	6%	0	NS
Electrical	27%	17%	NS

Demographics of the patients per group. Values are represented as mean ± standard deviation. IIT, intensive insulin treatment; TBSA, total body surface area; 3^rd^, percentage of total body surface area with 3^rd^-degree burn; NS, not significant (*P *-value > 0.05).

**Table 2:** Glucose values

Glucose value	Intensive insulin treatment	Exenatide	*P*-value
Six AM	123 ± 35 mg/dl	132 ± 27 mg/dl	0.39
Maximum	167 ± 45 mg/dl	171 ± 40 mg/dl	1.00
Minimum	98 ± 29 mg/dl	107 ± 26 mg/dl	0.09
Daily average	130 ± 28 mg/dl	138 ± 25 mg/dl	0.31

Average glucose values per group. Values are represented as mean ± standard deviation. NS, Not significant (*P- *value > 0.05).

**Figure 2:**
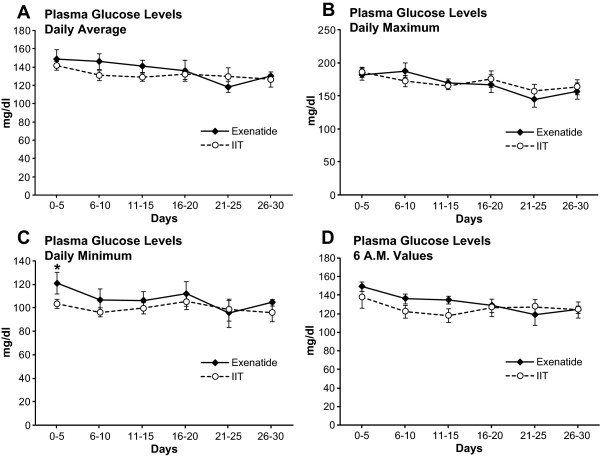
**Glucose values**. Longitudinal analysis of the different glucose values along the 30 days of study. If less than three glucose values were obtained, maximum and minimum values were not calculated (see text for details). **(a)** Daily average. **(b)** Maximum. **(c)** Minimum. **(d)** Six AM glucose levels. IIT: Intensive insulin treatment. All values are represented as mean ± SEM.

Longitudinal analysis of all the glucose values (6 AM, daily, maximum and minimum glucose levels) along the acute stay showed similar values in both groups (Figure 2). The incidence rate of days with less than three glucose determinations per acute stay was 15.5 days/30 days for the IIT group and 14.5 days/30 days in the exenatide group (*P *> 0.05).

**Figure 3:**
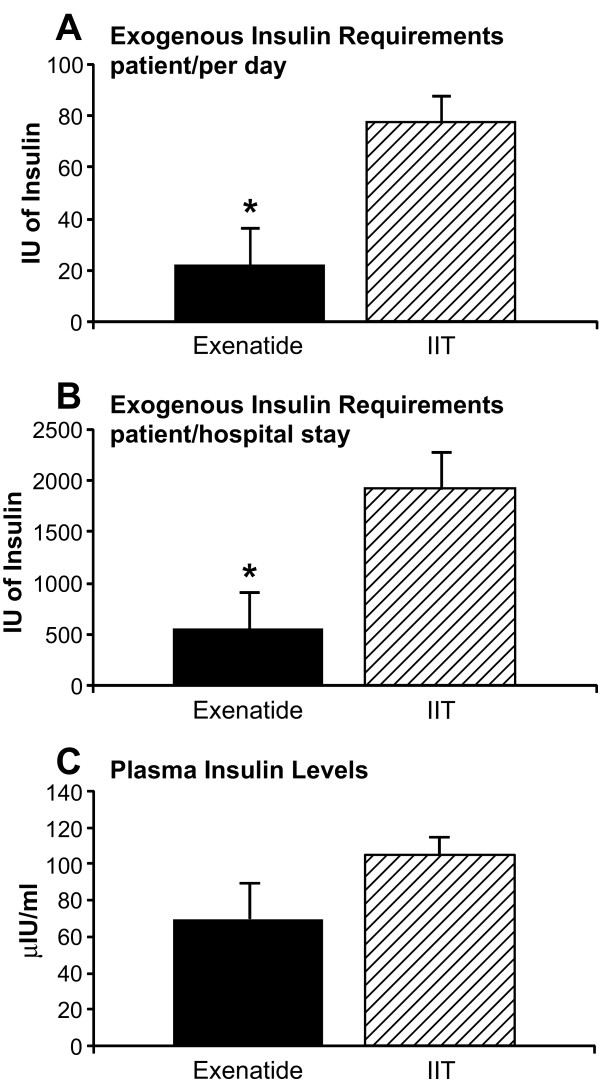
**Exogenous insulin administered and insulin plasma levels**. **(a)** Insulin administered per patient per day. **(b)** Average of administered insulin per patient per acute hospital stay. **(c)** Mean plasma insulin levels. All values account for first 30 days of hospital stay. Values are represented as mean ± SEM.

Patients in the IIT group received significantly more insulin to maintain similar glucose levels when compared to the exenatide patients (*P *= 0.01), while serum insulin levels (endogenous and exogenous) were not significantly different between both groups (*P *> 0.05) (Figure 3). Three patients in the exenatide group did not receive exogenous insulin, while all the patients in the IIT group received insulin.

**Figure 4:**
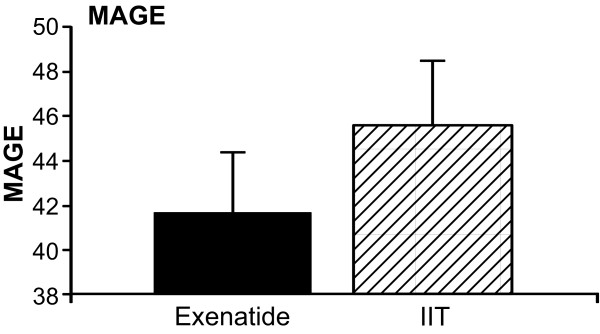
**MAGE**. Mean amplitude of glucose excursion. Expressed as mean ± SEM.

**Figure 5:**
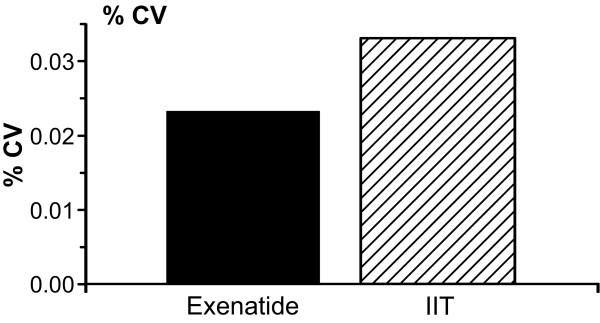
**Percentage of coefficient of variance (% CV)**.

The MAGE was not statistically different between both groups (*P *= 0.61) (Figure 4). Similarly, % CV was similar in both groups (Figure 5).

The incidence rate of hypoglycemia was similar in both groups (0.38 events/patient-month). There were 17 events of moderate hypoglycemia (40 to 59 mg/dl) and 1 of severe hypoglycemia (< 40 mg/dl) in the IIT group and 6 events of moderate hypoglycemia and none of severe hypoglycemia in the exenatide group.

**Figure 6:**
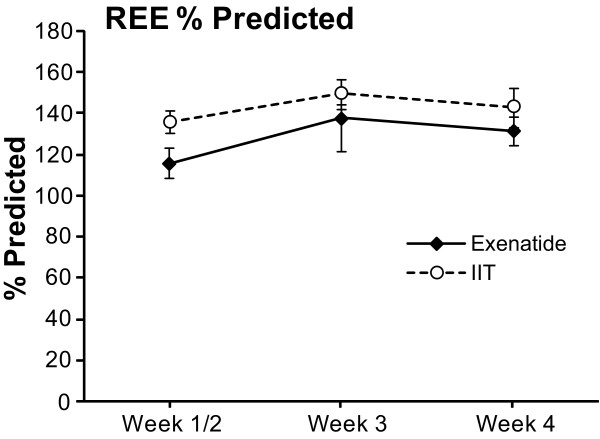
**Resting energy expenditure (REE)**. REE expressed as percentage of predicted. All values are represented as mean ± SEM.

**Figure 7:**
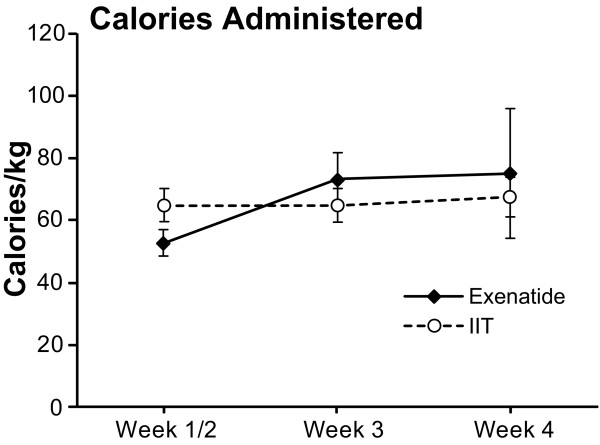
**Calories administered**. Total amount of calories administered (per Kg) to the patients during the acute stay (first 30 days from admission). Data expressed as mean ± SEM.

The REE [4] was not significantly different between the groups (Figure 6). The total amount of calories administered to the patients is shown in Figure 7. It was not different between groups.

Exenatide was well tolerated by all the patients and no adverse reaction associated with the administration of exenatide was documented. There was no mortality in any of the groups.

## Discussion

Incretin-based drugs are a family of substances that lower glucose levels by enhancing glucose-dependent secretion of insulin, a condition known as the incretin effect [24]. Incretin-based drugs require a hyperglycemic state for their effects [25], thus, hypoglycemia is uncommon even with high-doses of the drugs [26]. This and many other described effects of GLP-1 analogues, such as increased endogenous insulin secretion and peripheral glucose uptake and protection against ischemia/reperfusion, made these drugs particularly suitable for burn patients. Other drugs may decrease glucose levels with a low risk of hypoglycemia or may increase peripheral glucose uptake, but in theory with GLP-1 analogues we would achieve these effects with only one drug.

Among the incretin-based drugs, we decided to administer exenatide because it is known that it binds with equal affinity to the GLP-1 receptor and produces similar glucose-lowering actions to GLP-1 [27]. Additionally, exenatide is known to increase insulin-dependent glucose uptake in muscle and fat while GLP-1 does not have such action [28]. Furthermore, its longer half-life allows for subcutaneous administration q- 12 h facilitating the management of severely burned patients in the ICU. According to its described effects, we expected that patients receiving exenatide treatment would receive less exogenous insulin. Since hyperglycemia was treated with administration of exogenous insulin in both groups to achieve normal levels, we did not expect significantly different glucose levels between patients, as observed.

Variability of glucose has been associated with increased mortality and has been considered one of the most important goals of glucose management in ICU [29]. We, therefore, included analysis of MAGE. Although mean MAGE was lower in the patients receiving exenatide, with the actual sample size it is not possible to make any conclusion on this regard. We found no difference in the incidence of hypoglycemic events in both groups, probably as a result of the insulin administration with a target glucose level of 80 to 140 mg/dl in all patients. By modifying the target glucose level, we hypothesize that the incidence of hypoglycemia would decrease in these patients. Further studies are necessary to determine the safest target glucose that improves morbidity and mortality in the burn population.

This study was designed as an open-label pilot study and was one of the multiple trials we conduct at our institute. At our institute, we have several clinical trials to study the effect of anti-catabolic, anabolic, and glucose modulation agents. The primary aim of the present study was to assess efficacy and feasibility (that is, effects on insulin and glucose metabolism) in a limited patient population. Based on previous efficacy studies at our institute, we hypothesized that we would need about six patients in the intervention group. In order to decrease variability and decrease the low sample error, we randomized three times as many patients (18 patients) to the control group. We are clearly underpowered to draw any conclusions on the effect of GLP-1 on morbidity or mortality. However, we demonstrated in the present open label trial that GLP-1 appears to be a safe adjunct therapy to achieve glucose in a pediatric burn population.

Exenatide was originally described as a therapy for adult diabetic patients, and although its use in pediatric patients has been suggested [30,31], the available literature about its use and safety in these patients is scarce at best. It has been described that exenatide may cause some adverse effects that can be severe (for example, pancreatitis) [32]. However, we did not observe complications or any undesirable effect associated with exenatide administration in the studied patients (for example, gastrointestinal symptoms, injection site reactions and hypersensitivity).

This study has limitations due to the small number of patients and the fact that the treatment was not blinded for safety reasons. A larger sample size would be necessary to assess other aspects, such as true complication rate, variability of glucose and the effect of exenatide on hypoglycemia and REE. The percent predicted REE was lower (closer to normal) in the first two weeks post admission in the exenatide patients, although not statistically significant. The absorption of exenatide after subcutaneous administration might be inconsistent in edematous patients. It has been shown that oral glutamine increases circulating GLP-1 [33] potentially stimulating endogenous GLP-1 secretion in the IIT group. This might have affected the glucose metabolism in the IIT group. Plasma concentration of exenatide and GLP-1 should be included in future studies. It would have also been interesting to determine C-peptide in plasma to assess endogenous insulin production and glucagon concentrations and we recommend doing so for future studies. However, this study was the first attempt to find alternate glucose lowering agents in the setting of burn critical care. We believe that the utility of alternative drugs, (for example, peroxisome proliferator-activated receptors (PPAR) agonists), needs to be assessed since many of these agents are less expensive and as equally safe as GLP-1. Therefore, a study assessing the utility of these drugs in the burn population is warranted. In this trial, we found that exenatide was effective, safe, and well tolerated by the severely burned pediatric population.

## Conclusions

Administration of exenatide to severely burned pediatric patients reduced the amount of administered exogenous insulin and was well tolerated during their acute setting. The GLP-1 analogue tested in this trial appears to be safe and reliably modulates glucose in these patients.

## Key messages

• The administration of exenatide resulted in a reduced amount of administered exogenous insulin in severely burn pediatric patients.

• Exenatide appears to be a safe and reliable glucose modulator in these patients.

## Abbreviations

BMI: body mass index; ELISA: enzyme-linked immunosorbent assay; GIP: glucose-dependent insulinotropic peptide; GLP-1: glucagon-like peptide-1; HbA1C: glycosylated hemoglobin; ICU: intensive care unit; IIT: intensive insulin treatment; IU: international units; MAGE: mean amplitude of glucose excursions; % CV: percentage of coefficient of variance; PO: per os; PPAR: peroxisome proliferator-activated receptors; REE: resting energy expenditure; SD: standard deviation; SEM: standard error of measurement; TBSA: total body surface area; TEN: total enteral nutrition.

## Competing interests

The authors declare that they have no competing interests.

## Authors' contributions

GAM, DNH, GAK, NCB, AMA, RK, HGR, FNW, LKB and MGJ were involved in study conception, design, data acquisition, analysis, and manuscript drafting. MGJ was involved in editing and final approval of the manuscript. All authors read and approved the final manuscript.
